# The effect of breaking up prolonged sitting on paired associative stimulation-induced plasticity

**DOI:** 10.1007/s00221-020-05866-z

**Published:** 2020-08-28

**Authors:** E. Bojsen-Møller, M. M. Ekblom, O. Tarassova, D. W. Dunstan, O. Ekblom

**Affiliations:** 1 The Swedish School of Sport and Health Sciences, GIH, 11486 Stockholm, Sweden; 2 Department of Neuroscience, Karolinska Institutet, Stockholm, Sweden; 3 Baker Heart and Diabetes Institute, Melbourne, VIC Australia; 4 Mary MacKillop Institute of Health Research, Australian Catholic University, Melbourne, VIC Australia

**Keywords:** Sedentary behaviour, Paired associative stimulation, Corticospinal excitability, Transcranial magnetic stimulation

## Abstract

Paired associative stimulation (PAS) can induce plasticity in the motor cortex, as measured by changes in corticospinal excitability (CSE). This effect is attenuated in older and less active individuals. Although a single bout of exercise enhances PAS-induced plasticity in young, physically inactive adults, it is not yet known if physical activity interventions affect PAS-induced neuroplasticity in middle-aged inactive individuals. Sixteen inactive middle-aged office workers participated in a randomized cross-over design investigating how CSE and short-interval intracortical inhibition (SICI) were affected by PAS preceded by 3 h of sitting (SIT), 3 h of sitting interrupted every 30 min by 3 min of frequent short bouts of physical activity (FPA) and 2.5 h of sitting followed by 25 min of moderate-intensity exercise (EXE). Transcranial magnetic stimulation was applied over the primary motor cortex (M1) of the dominant abductor pollicis brevis to induce recruitment curves before and 5 min and 30 min post-PAS. Linear mixed models were used to compare changes in CSE using time and condition as fixed effects and subjects as random effects. There was a main effect of time on CSE and planned within-condition comparisons showed that CSE was significantly increased from baseline to 5 min and 30 min post-PAS, in the FPA condition, with no significant changes in the SIT or EXE conditions. SICI decreased from baseline to 5 min post-PAS, but this was not related to changes in CSE. Our findings suggest that in middle-aged inactive adults, FPAs may promote corticospinal neuroplasticity. Possible mechanisms are discussed.

**Electronic supplementary material:**

The online version of this article (10.1007/s00221-020-05866-z) contains supplementary material, which is available to authorized users.

## Introduction

Neuroplasticity refers to the ability of the nervous system to undergo enduring morphological or functional change in response to the demands of its environment. In animal models, regular physical activity initiates cellular and molecular processes related to neuroplasticity (Cotman and Berchtold 2002), leading to improvements in learning and memory (van Praag et al. 1999). In humans, non-invasive transcranial magnetic stimulation (TMS) is often used to probe corticospinal adaptations. Paired associative stimulation (PAS) can induce an effect similar to long-term potentiation, a mechanism of neuroplasticity that enhances synaptic communication, within the human corticospinal system (Stefan et al. 2000). As such, the effects of PAS on corticospinal excitability has been used as a measure of the propensity for neuroplasticity. Habitually more physically active subjects show a larger effect of PAS as compared to their less active counterparts (Cirillo et al. 2009). In addition, an acute bout of moderate-intensity exercise has been shown to improve retention of a motor task (Roig et al. 2012) and enhance the effect of PAS compared to rest (Singh et al. 2014b). Thus, it seems that both acute and long-term physical activity enhances neuroplasticity.

Among young physically active adults, moderate-to-vigorous physical activity has been shown to enhance corticospinal neuroplasticity (Mang et al. 2014; Singh et al. 2014b; Lulic et al. 2017) and reduce short-interval intracortical inhibition (SICI) (Singh et al. 2014a; Smith et al. 2014; Mooney et al. 2016), which is thought to directly influence cortical excitability (Kujirai et al. 1993). Office-work is often characterized by prolonged periods of sitting and inactivity. However, it is not known to what extent prolonged sitting influences processes of corticospinal neuroplasticity. While it has been demonstrated that physically inactive individuals are less responsive to PAS (Cirillo et al. 2009), it is not known to what degree physical activity interventions modulate corticospinal neuroplasticity in inactive middle-aged adults (e.g. office workers). McDonnell et al. (2013) found that a 30-min exercise at low intensity was more effective than a 15-min exercise at a moderate intensity (55% versus 77% of age predicted maximal heart rate) for promoting neuroplasticity in a mixed healthy mixed age population. Not all office workers can take time off during the work day for exercise, and previous research has suggested that reminders for breaking up sedentary behaviour at the office desk is desired by office workers (Nooijen et al. 2018). Breaking sedentary behaviour at the desk can be feasibly done with the use of frequent short bouts of physical activity (Climie et al. 2018; Larsen et al. 2019). In this randomized cross-over study, we assessed the propensity for neuroplasticity in sedentary middle-aged office workers undergoing acute exposure to three distinct ecologically valid physical activity patterns. We used PAS to induce corticospinal neuroplasticity and assessed changes in excitability with single-pulse TMS. Specifically, we assessed the extent to which prolonged sitting (SIT), sitting interrupted by low-intensity frequent short bouts of physical activity (FPA), and prolonged sitting followed by a bout of moderate-intensity aerobic exercise (EXE), in combination with a PAS intervention influences corticospinal excitability (CSE) in middle-age sedentary adults. We also used a paired TMS protocol to assess if changes in SICI could be a mechanism for how day-to-day variations in activity patterns might change CSE.

While in young active individuals, EXE was shown to improve corticospinal long-term potentiation-like plasticity, this is the first investigation to assess impacts of breaking up sedentary behaviour with FPA. Breaking up prolonged sitting with FPA has been previously shown to improve glucose regulation (Dunstan et al. 2012) and cognitive performance including working memory and attention (Mullane et al. 2016). We, therefore, hypothesised that

1. The EXE condition would result in larger increases in CSE and decreases in SICI as compared to both the SIT and the FPA.
2. The FPA would result in larger increases in CSE after the PAS intervention compared to the SIT.

## Methods

### Participants

Inactive middle-aged participants were recruited through email invitations to office employees at two collaborating companies and via public advertising. Participants were excluded if they had a BMI above 35 kg/m^2^, participated in more than 150 min physical activity per week, or had diabetes or any history of cardiovascular disease. After providing informed consent, 16 healthy working age (mean 52.6 ± 8.1 years; see Table 1 for subject characteristics) participants were enrolled in the study. All participants reported no use of medication and were screened with the TMS adult safety screening tool (TASS) (Keel et al. 2000) for any contraindication to TMS. The study was conducted in accordance with the Declaration of Helsinki and was approved by the Regional Ethical Review board (2016//2096-31 and 2017/198).

**Table 1:** Subject characteristics

	Min	Max	Mean (SD)
Age (years)	42	70	52.6 (8.1)
*V*O_2max_ (ml/kg/min)	21.2	40.6	31.3 (6.2)
*V*O_2max_ (l/min)	1.9	3.5	2.6 (0.5)
Length (cm)	160	193	175.2 (8.0)
Waist circumference (cm)	85.0	119.2	101.5 (10.8)
Body mass (kg)	59.5	111.6	82.8 (12.4)
% maximal heart rate at exercise bout	53.3	87.2	73.4 (8.7)

*VO*_*2max*_ estimated maximal oxygen consumption

### Study design

**Fig. 1:**
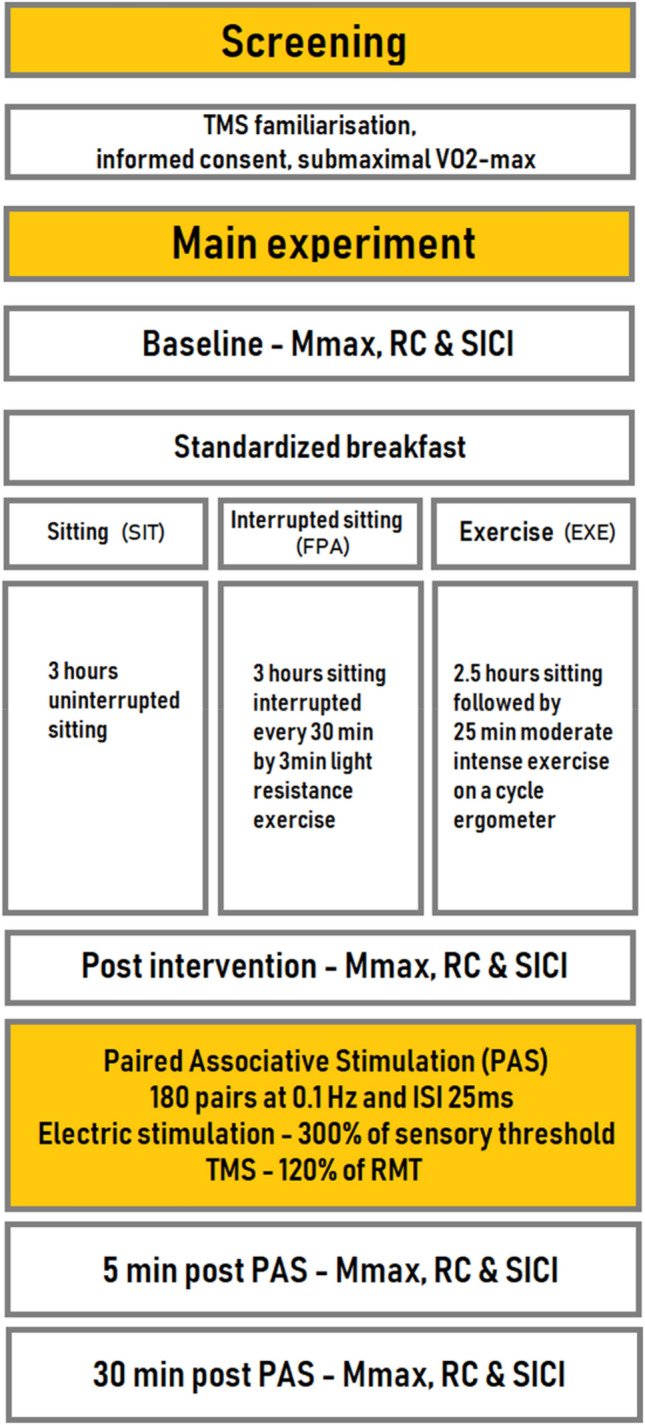
An overview of the experimental protocol. In a cross-over design, participants underwent three experimental conditions with a wash-out period of at least 7 days. *VO*_*2max*_ estimated maximal oxygen consumption, *Mmax* maximal compound muscle action potential, *RC* recruitment curve, *SICI *short-interval intracortical inhibition

Figure 1 displays the experimental design of the study. All participants attended the laboratory on four separate occasions consisting of one screening session and three experimental sessions in a block-randomized cross-over design. On the day of the screening session anthropometrics, a thorough TMS-familiarization and a submaximal oxygen consumption test used to estimate maximal oxygen consumption (*V*O_2max_) were carried out. Each condition was preceded by a 4-day run-in period. All experimental conditions were separated by a wash-out period of at least 7 days.

#### Run-in period

Four days prior to each experimental session, participants were equipped with a hip-worn accelerometer (ActiGraph, GT3X) for the measurement of physical activity and sedentary behaviour. Furthermore, participants filled in an activity and sleep diary and were instructed not to exercise the day prior to each experimental condition, but to maintain their habitual activity level. On the day prior to each condition they were asked to record their food intake and to consume a standardized dinner (i.e. the same meal was given at the three time points) no later than 8:00 pm. Participants were also asked to fast and only drink water from 8:00 pm on the evening before each condition until approximately 8.30 am when a standardized breakfast was served in the laboratory. The amount of each ingredient were kept as identical as possible, i.e. to repeat the same status for a participant during the test day. Hence, no attempt was made to standardize amount of calories or macronutrients from body mass or estimated resting energy consumption.

#### Day of the experimental condition

To avoid any physical activity prior to the experiment, participants were transported to the laboratory by a cab from their home address. Upon arrival, participants returned the sleep and activity diary. Thereafter, TMS preparation was carried out, including hotspot detection and other electrophysiological measurements (for more details, see TMS procedure). After breakfast, the 3-h experimental period began. Immediately after each condition, TMS measurements were obtained. Thereafter, a PAS intervention was carried out. Five minutes and 30 min after the PAS protocol, TMS measurements were obtained.

#### Experimental conditions

The prolonged sitting condition (SIT) consisted of 3 h of uninterrupted sitting. Every 30th min, a test leader entered the room for a 3-min social break. The frequent short bouts of physical activity (FPA) condition consisted of 3 h of sitting interrupted every 30th min by 3 min of simple physical activities (see below). The exercise condition (EXE) consisted of 2.5 h of sitting followed by a 25-min moderate-intensity exercise bout (see below for details). Participants were allowed to read in a book, but were not allowed to watch television, use mobile phones or tablet during the sitting period. Sleeping was not permitted.

Each 3-min FPA bout consisted of 3 rounds of 3 separate simple exercises performed for 20 s each (total—3 min). Participants watched a video of the exercises and were instructed to follow the pace of the exercises shown in the video. The video and exercises was adopted from Dempsey et al. (2017). The first exercise was a box squat standardized to a depth of 90° knee flexion. The second exercise was calf raises. The third exercise was a gluteus maximus contraction followed by a knee raise.

Prior to the exercise bout participants were equipped with a chest heart rate monitor (Polar H10, Polar Electro, Kempele, Finland). The exercise bout of the EXE experimental condition consisted of 25-min exercise on a cycle ergometer (Monark model 828E, Varberg, Sweden) at a moderate intensity that corresponded to 12 at the Borg’s scale and with an individual selected cadence. Heart rate was obtained as a 1-min average every 5th min throughout the exercise bout and a total mean for the whole exercise bout was calculated for each participants and is reported as percentage of estimated maximum heart rate (220-age) in Table 1.

### TMS procedure

Participants were seated in a chair and the m. abductor pollicis brevis (APB) was prepared for surface electromyography (EMG). Two electrodes (BlueSensor, Ambu, Ballerup, Denmark) were placed over the muscle belly in a bipolar montage with a inter electrode distance of 1 mm. A ground electrode was placed at the head of the first metacarpal. EMG was amplified (1000×), band pass filtered (10 Hz–2.5 kHz) and sampled at 5 kHz (CED 1401+). The arm rested on a pillow placed in the participants´ lap. Signal-to-noise ratio was inspected with a maximal contraction of the APB using Spike 2 software (version 7, Cambridge Electronic Design Ltd., UK).

A four-camera motion capture system (Oqus 7, Qualisys AB, Gothenburg, Sweden) with reflective markers placed on the head and coil was used to locate the subjects’ head and the coil in three-dimensional space. Continuous kinematic data were collected at 250 Hz using Qualisys Track Manager (QTM, version 2.14, Qualisys AB, Gothenburg, Sweden) and synchronously transferred to MATLAB (version R2015b, The MathWorks, Inc., USA) software using MATLAB plug-in for QTM (version 1.12, Qualisys AB, Gothenburg, Sweden). Further, the relative distance between the coil and the participant head was calculated frame-by-frame using script written in MATLAB. When the hotspot was identified, the positioning of the coil relative to the head position was saved and used as a reference point. During all measurements, the researcher was provided with continuous visual feedback of any linear or angular displacement of the coil from the reference point (hotspot), to enhance precision.

All TMS stimulations were applied to the M1 contralateral to the dominant hand using a figure-of-eight coil. Two Magstim 200 stimulators (Magstim Company Ltd., Whitland, Wales, UK) coupled by a bi-stim module were connected to a computer that triggered all stimulations through a CED 1401+ and Signal 6.04a software (Cambridge Electronic Design Ltd., UK). To induce a posterior-to-anterior current in the hand knob of the M1, the coil was placed on the scalp at a 45° angle to the mid-sagittal plane, during all measurements.

A mini-mapping procedure was used to locate the APB hotspot. The hotspot was identified as the location whereby the largest and most consistent motor evoked potential (MEP) appeared using a supra threshold stimulation intensity. Resting motor threshold (RMT) was defined as the lowest intensity that elicited a MEP with a peak-to-peak amplitude of above 50 µV in the APB EMG for at least five out of ten stimulations. The maximal compound muscle action potential (*M*_max_) of the APB was obtained prior to every TMS measurement. Peripheral electric stimulations were applied to the median nerve through a bipolar montage of two electrodes (BlueSensor, Ambu, Ballerup, Denmark). A constant current stimulation (Digitimer model DS7A, Digitimer, UK) was used to induce 0.5-ms pulses at an inter-stimulus interval (ISI) of 4 s. The intensity of the current was increased until the maximal M-wave, measured as peak-to-peak amplitude, was obtained. Once an increase in stimulus intensity did not result in a further increase in the M-wave, a further 10% increase in intensity was applied to ensure the maximal M-wave was obtained.

**Fig. 2:**
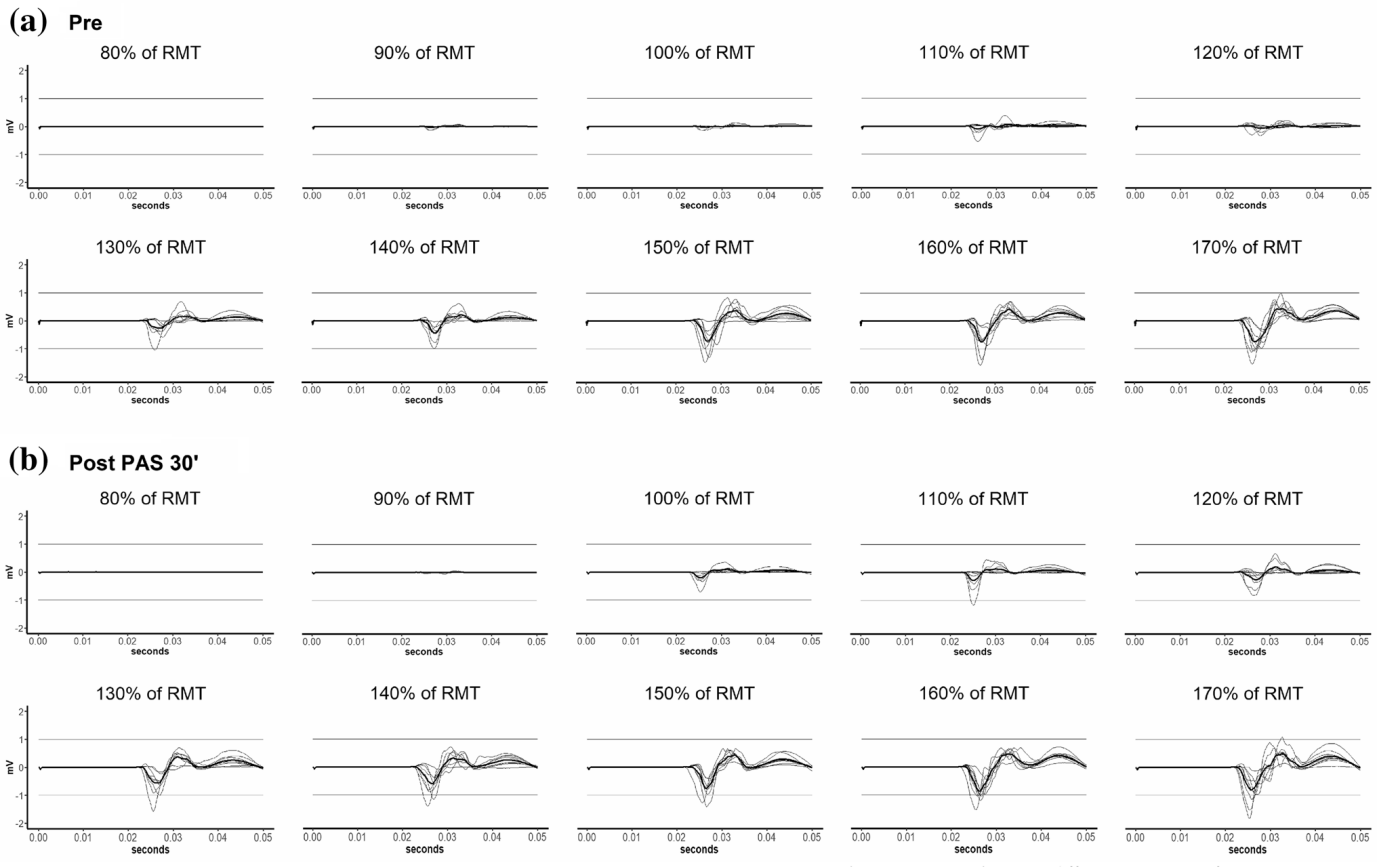
Raw abductor pollicis brevis motor evoked potentials (MEPs) in response to motor cortex transcranial magnetic stimulation at different intensities for one representative subject before and 30 min after paired associative stimulation (PAS) in the frequent short bout of physical activity (FPA) experimental condition. Recruitment curves **a** at baseline and **b** Post-PAS 30 min are shown with all motor evoked potentials presented as thin lines. Mean of all MEPs within a given stimulation intensity are displayed as a thick black line. Stimulation intensities are expressed in percent of resting motor threshold (% of RMT) ranging from 80% RMT to 170% RMT

The MEP recruitment curves (RC) were acquired at baseline, after the experimental condition and 5 min and 30 min post-PAS (see Fig. 1), using intensities in 10% steps ranging from 80 to 170% of RMT. The RC consisted of 80 single-pulse TMS stimulations applied in random order. Figure 2 displays raw MEPs of one representative participant.

After each RC, SICI was measured using a conditioning stimulation at 80% of RMT and a test stimulation at 140% of RMT with a 3-ms interval between the paired pulses. Ten conditioned pairs of stimulation and ten test stimulations were applied in random order at each time point.

#### Paired associative stimulation (PAS)

After each experimental condition, the PAS protocol (Stefan et al. 2000) was applied. The protocol consisted of 180 pairs of peripheral electric and single-pulse TMS stimulations delivered at 0.1 Hz. The intensity of the peripheral electric stimulation was defined as three times above sensory threshold. The intensity of the TMS stimulations was 120% of RMT. Participants were instructed to count stimulations and direct their attention to the hand for the duration of the protocol, because attention has been shown to modulate the effect of PAS (Stefan et al. 2004).

### Physical activity measurement

Time spent in different physical activity behaviours was objectively measured using a hip-worn accelerometer (ActiGraph model GT3X, Pensacola, FL, US). Variables obtained were % of time spent in sedentary behaviour and moderate-to-vigorous physical activity. The accelerometers were worn for 4 days prior to the experiment and was only carried during waking hours. Accelerometers were initialized and downloaded using the software Actilife (version 6.13.3, ActiGraph, Pensacola, FL, US). The tri-axial vector magnitude signal was sampled at 30 Hz downloaded and turned into counts. The signal was then down sampled into epochs of 60 s. The intensity of physical activity was presented as counts per minutes. Sedentary time was defined as count below a threshold for 200 counts per minutes (Sasaki et al. 2011). Moderate-to-vigorous physical activity was defined as activities eliciting at least 2690 counts per minutes (Sasaki et al. 2011). Drop time was allowed for maximal 2 min. Days with at least 10 h of wear time were considered valid and participants with at least 3 valid days were included.

### Submaximal *V*O_2max_

To estimate maximal oxygen consumption, an Ekblom-Bak submaximal *V*O_2max_ test (Ekblom-Bak et al. 2014) was carried out on a cycle ergometer (Monark, model 828E, Varberg, Sweden). Changes in heart rate (∆HR) and power output (∆PO) between a standardized lower work load and an individualized higher work load and the ratio (∆HR/∆PO) between those two was calculated. Age, the ∆HR/∆PO ratio, the ∆PO and the mean heart rate at the standard work rate were all entered into the gender-specific equation developed by Bjorkman et al. (2016) to estimate *V*O_2max_ for each participant.

### Data analysis

#### Evoked potential analysis

The APB EMG response associated with each TMS stimulation was inspected offline. Stimulations with more than two times the root mean square amplitude of the standard noise 50 ms prior to the TMS stimulation were discarded. For all TMS measures, peak-to-peak amplitude of the APB MEP was obtained and normalized to the size of the *M*_max_. Area under RC (AURC) was calculated using the trapezoidal method (Purves 1992), which relies on linear interpolation between two data points to estimate AURC. AURC is calculated as the average MEP size between two stimulation intensities times the difference in stimulation intensity. SICI consisted of ten paired-pulse stimulations and ten test stimulations. A mean was calculated for each condition and time point. SICI was calculated as the ratio between the paired-pulse (conditioned) mean and the test (1.4 RMT) mean. In a sensitivity analysis, all trials with MEP amplitudes ± 2 standard deviations for every given intensity were discarded before the mean was calculated. This was done for all TMS measures.

#### Statistics

All statistics were carried out in R-studio software. Distribution of the variables was investigated using Shapiro–Wilk’s test of normality. Variables that violated the assumption of normality were log-transformed to the base of 10 so that normality was obtained. Normality tests were repeated after transformation to confirm that normality of the data was achieved. Linear mixed models were fitted using the lme4 package described by Bates et al. (2015). Specific multiple comparisons were calculated using the Multcomp package described by Hothorn et al. (2008). Level of statistical significance was set to *p* < 0.05. Outcome variables were entered into the linear mixed model with time (four levels) and condition (three levels) as fixed effects and subjects entered as random effects. Only predefined pairwise comparisons were carried out. Within-condition predefined pairwise comparisons were defined to compare baseline values to all other time points. Post-condition responses were compared to post-PAS 5 min and post-PAS 30 min and post-PAS 5 min were compared to post-PAS 30 min. Furthermore, between conditions predefined pairwise comparisons were carried out at each time point. All *p *values were adjusted according to the number of comparisons in each models, using the Holm–Sidak method of adjustment. To test the relationship between SICI and AURC at baseline, bivariate correlations were performed. Furthermore, bivariate correlation between changes in AURC and changes in SICI was carried out.

## Results

Table 2 displays the electrophysiological measurements at baseline for the three conditions. Table 3 shows RC MEPs in millivolt at baseline in the three different conditions (for supplemental material of the statistical analysis, please see Online Resource File 1, Tables 4 and 5).

**Table 2:** Baseline measures in the three conditions

	SIT	FPA	EXE
	Mean (SD)	Mean (SD)	Mean (SD)
RMT (%)	34.87 (8.16)	35.31 (8.32)	34.44 (7.99)
Mmax_Pre_ (mV)	4.23 (0.77)	3.93 (1.20)	4.40 (0.65)
AURC_Pre_	14.73 (10.87)	13.99 (9.66)	14.81 (8.59)
SICI_MEP.uncond.pre_ (mV)	1.24 (0.97)	1.01 (0.94)	1.08 (0.61)
SICI_MEP.cond.pre_ (mV)	0.14 (0.12)	0.12 (0.09)	0.13 (0.10)
SB_prior_ (%)	61.26 (11.49)	60.01 (10.06)	59.51 (12.46)
MVPA_prior_ (%)	5.58 (3.35)	5.68 (3.11)	5.57 (3.58)

*RMT* resting motor threshold in % of maximal stimulator output, *Mmax*_*pre*_ maximal peak-to-peak amplitude of the compound muscle action potential at baseline, *AURC*_*pre*_ area under the recruitment curve at baseline. *SICI*_*MEP.uncond.pre*_ peak-to-peak amplitude of the raw MEP evoked using a TMS stimulus output of 140% RMT without conditioning stimulation at baseline. *SICI*_*MEP.cond.pre*_ peak-to-peak amplitude of the raw MEP evoked using a TMS stimulus output of 140% RMT with a conditioning stimulation at 80% RMT delivered at an inter-stimulus interval of 3 ms at baseline. *SB*_*prior*_ % sedentary behaviour on the day prior to the session, *MVPA*_*prior*_ % moderate-to-vigorous physical activity performed on the day prior to the session

**Table 3:** Baseline mean peak-to-peak amplitude in millivolt of motor evoked potentials elicited by stimulations at different intensities

% of RMT	SIT	FPA	EXE
80	0.02 (0.01)	0.02 (0.01)	0.03 (0.04)
90	0.05 (0.06)	0.03 (0.03)	0.09 (0.07)
100	0.18 (0.15)	0.15 (0.19)	0.20 (0.20)
110	0.39 (0.33)	0.40 (0.34)	0.43 (0.39)
120	0.70 (0.56)	0.59 (0.56)	0.72 (0.51)
130	0.97 (0.87)	0.74 (0.63)	0.97 (0.62)
140	1.17 (1.00)	0.88 (0.76)	1.13 (0.73)
150	1.33 (1.04)	1.02 (0.75)	1.25 (0.70)
160	1.30 (1.03)	1.06 (0.76)	1.35 (0.65)
170	1.41 (1.16)	1.17 (0.74)	1.27 (0.74)

### Effects on CSE

**Fig. 3:**
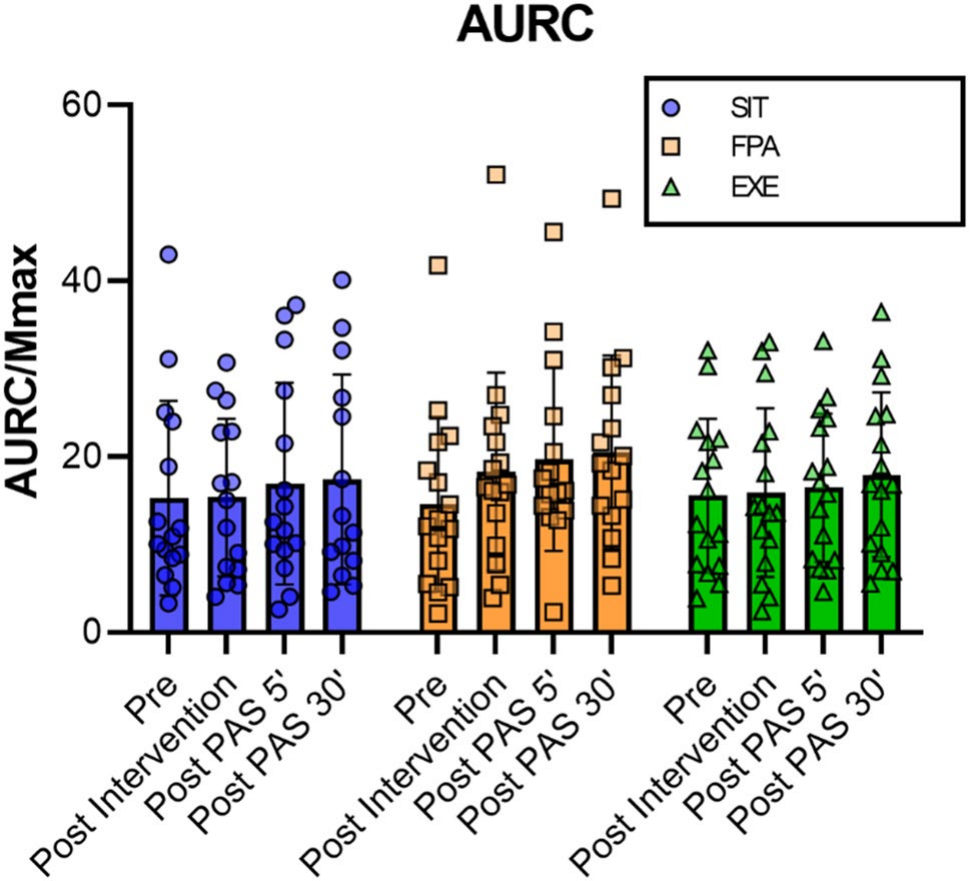
Area under the recruitment curve for each condition. SIT (blue) = sitting condition marked with open circle. FPA (orange) = frequent short bout of physical activity condition marked with open square. EXE (green) = exercise condition marked with open triangle. Each symbol illustrates the value of one subject. The height of the bars represent the mean ± SEM

Figure 3 shows the changes in AURC over the course of the experiment for each condition. There was a main effect of time on AURC (*p* = 0.002), but the linear mixed model revealed no main effect of condition (*p* = 0.113) and no interaction between time and condition on AURC (*p* = 0.500).

Specific multiple comparisons for the effect of time revealed that AURC increased significantly from baseline to 30 min after PAS (*β* = − 0.113, SE = 0.030, *p* = 0.001).

Pre-planned, within-condition comparisons revealed that the AURC increased from baseline to 5 min (*β* = − 0.158, SE = 0.051, *p* = 0.037) and 30 min (*β* = − 0.184, SE = 0.051, *p* = 0.006) after PAS, only in the FPA condition. All other comparisons were not statistically significant (*p* > 0.233).

There was no difference between conditions in AURC at baseline (*p* > 0.118), post-intervention (*p* > 0.118), 5 min (*p* > 0.071) and at 30 min after PAS (*p* = 0.150).

### Effects on SICI

A linear mixed model showed that there was no interaction between time and condition on SICI (*p* = 0.577) and no main effect of condition (*p* = 0.440), but a main effect of time (*p* = 0.007).

Specific multiple comparisons for the effect of time showed that SICI significantly decreased from baseline to 5 min after PAS (*β* = − 0.153, SE = 0.045, *p* = 0.037). There were no other significant differences between any time points (*p* > 0.075). Pre-planned within-condition analysis showed no significant changes in SICI within any group at any time point (*p* > 0.226).

### Associations between SICI and AURC

There were no significant associations between baseline SICI and AURC (*p* > 0.209) and also no associations between changes in SICI and AURC (*p* > 0.151).

### *M*_max_

Changes in *M*_max_ were investigated to examine whether AURC alterations were driven by changes in *M*_max_. Specific multiple comparisons between *M*_max_ at baseline, after the intervention and after PAS revealed a tendency towards a reduction in *M*_max_ from baseline to 5 min after PAS in the exercise condition only (*p* = 0.073). All other comparisons showed no changes in *M*_max_ (*p* > 0.740). *M*_max_ did not differ between conditions at baseline (*p* > 0.388) (see Table 2).

### Sensitivity analysis

Sensitivity analyses were carried out discarding all trials with MEP amplitudes ± 2 standard deviations. This did not affect the results of the study.

## Discussion

The present study investigated the extent to which neuroplasticity of CSE and SICI is influenced by different physical activity patterns. There was an effect of time on CSE, suggesting that the PAS protocol enhanced plasticity in all conditions. However, exploratory pre-planned within-condition analyses revealed that CSE significantly increased in the FPA condition, while no such increase was seen in the SIT or EXE condition. These alterations in AURC were not related to changes in SICI.

### Effects of PAS on CSE and SICI in a middle-aged inactive population

In our middle-aged sample, there was a significant increase in CSE as measured via AURC at 30 min after PAS as compared to baseline. The effect of the PAS protocol has previously been shown to be reduced as a function of age (Müller-Dahlhaus et al. 2008). In addition, level of physical activity has been shown to influence the effect of the PAS protocol with highly active subjects showing stronger effects of PAS as compared to inactive subjects (Cirillo et al. 2009). The participants in the current study were inactive and had low fitness level (see Table 2) (Gupta et al. 2020). Thus, large effects of the PAS protocol were not expected. Still, our findings support that PAS, under certain circumstances, can induce increases in CSE, in a middle-aged inactive population.

### Effects of physical activity condition on changes in CSE and SICI

In the present study, there was no interaction between time and condition for either CSE or SICI. However, our pre-planned analysis for CSE showed that there was a significant increase from baseline to both 5 min and 30 min post-PAS in the FPA condition. There were no such increases in the SIT or in the EXE condition. Possible mechanisms are discussed below.

#### Possible mechanisms for the findings

Previous research has shown that an acute bout of either moderate- (Singh et al. 2014b) or high-intensity (Mang et al. 2014) aerobic exercise prior to PAS significantly increases excitability (see (Mellow et al. 2019) for review), but we did not see such effect in the present study. As in previous literature, the present study used rate of perceived exertion to set the intensity of the exercise bout. The population investigated in the present study may have rated the intensity differently than a young active population. However, in the present study, the mean (± SD) heart rate was 73.4 (± 8.7) % of age estimated maximal heart rate, compared to a target heart rate of 65–70% of age estimated maximal heart rate in Singh et al. (2014b). In addition, McDonnell et al. (2013) reported that, low-intensity cycling induced long-term depression-like neuroplasticity, whereas rest or moderate-intensity exercise did not. This is in line with the findings in the present study.

The effect of PAS is sensitive to attention (Stefan et al. 2004). Low levels of attention have been shown to abolish the effect of PAS (Stefan et al. 2004). Breaking up long periods of sitting has been shown to enhance cognitive performance (Mullane et al. 2016) including a working memory test that depends on attention. While many other mechanisms are possible, the FPA condition may have improved attention, and thereby increased the effect of PAS.

In addition to the effects on attention, there is consistent evidence that replacing sedentary time with light-intensity physical activity enhances postprandial metabolic parameters (Benatti and Ried-Larsen 2015). Breaking up prolonged periods of sitting with brief periods of FPA has been shown to improve blood glucose regulation (Dunstan et al. 2012), decrease insulin levels and increase insulin sensitivity (Duvivier et al. 2017) in overweight/obese adults. However, it is not known whether the glucose regulation in the brain is affected to the same extent. More research is needed to understand the mechanisms of glucose regulation in relation to neuroplasticity.

### Associations of changes in CSE and SICI

We found no association between changes in CSE and SICI. In contrast, SICI has previously been shown to be reduced after an acute bout of aerobic exercise (Smith et al. 2014) and after aerobic exercise in combination with PAS (Singh et al. 2014b). In the study of Singh et al., the test stimulus intensity was reset to evoke a MEP of 1 mV at each time point, while in our study the test stimulus intensity was set and kept throughout the protocol. This difference in SICI methodology might explain some of the differences in findings between studies. In the present study, there was a significant decrease in SICI from baseline to 5 min after PAS, regardless of condition, but this difference was absent 30 min post-PAS. Several other studies have not detected changes in SICI after plasticity-inducing protocols (Ridding and Taylor 2001; Stefan et al. 2002). Thus, the changes in SICI shown here are unlikely to be mediated by PAS alone.

### Strengths and limitations

A major strength with this study is the highly controlled experimental conditions combined with standardized run-in periods allowing us to investigate day-to-day changes in corticospinal neuroplasticity within the same individuals. A limitation of the current study was that the application of PAS to the same subject of three different days could possibly have induced a history effect in CSE. However, such history effect is unlikely to have affected our results since the order of conditions was counterbalanced and randomized between subjects. Furthermore, there was no difference between baseline resting thresholds between the first 2nd and 3rd sessions. Large intraindividual variability has been shown when two PAS protocols were applied on different days (Fratello et al. 2006). Our exploratory finding that FPA may promote neuroplasticity suggests that some of the previously reported variability could have been caused by poor standardization of physical activity undertaken by the subject in the hours prior to the investigation.

A limitation of the current study was that we did not describe the genetic profile of the participants. The Val66Met polymorphism on the BDNF-gene has been shown to influence levels of corticospinal plasticity (Cheeran et al. 2009). It has been shown that increases in MEP amplitudes after motor learning are decreased in Met-carriers compared to Val-carriers (Kleim et al. 2006). The effect of PAS has also been shown to rely on the COMT polymorphism (Witte et al. 2012). Future investigations might build upon ours by investigating activity-related day-to-day variations in CSE and corticospinal plasticity in active and inactive individuals of different ages with different genetic profiles.

## Conclusion

In the present study, we demonstrate that PAS induced increases in CSE in inactive middle-age adults regardless of preceding physical activity pattern. However, pre-planned analysis showed that this increase in excitability was driven by the FPA condition, suggesting that FPA may promote neuroplasticity. SICI was decreased at 5 min post-PAS when conditions were collapsed, but this was not related to changes in AURC. The question of what psychological and/or neurophysiological mechanisms modulate corticospinal neuroplasticity in inactive middle-aged adults awaits further experimental scrutiny.

## Electronic supplementary material

Below is the link to the electronic supplementary material.

Supplementary file1 (DOCX 13 kb)

## References

[CR1] Bates D, Mächler M, Bolker B, Walker S (2015). Fitting linear mixed-effects models Usinglme4. J Stat Softw.

[CR2] Benatti FB, Ried-Larsen M (2015). The effects of breaking up prolonged sitting time: a review of experimental studies. Med Sci Sports Exerc.

[CR3] Bjorkman F, Ekblom-Bak E, Ekblom O, Ekblom B (2016). Validity of the revised Ekblom Bak cycle ergometer test in adults. Eur J Appl Physiol.

[CR4] Cheeran BJ, Ritter C, Rothwell JC, Siebner HR (2009). Mapping genetic influences on the corticospinal motor system in humans. Neuroscience.

[CR5] Cirillo J, Lavender AP, Ridding MC, Semmler JG (2009). Motor cortex plasticity induced by paired associative stimulation is enhanced in physically active individuals. J Physiol.

[CR6] Climie RE, Wheeler MJ, Grace M (2018). Simple intermittent resistance activity mitigates the detrimental effect of prolonged unbroken sitting on arterial function in overweight and obese adults. J Appl Physiol (1985).

[CR7] Cotman CW, Berchtold NC (2002). Exercise: a behavioral intervention to enhance brain health and plasticity. Trends Neurosci.

[CR8] Dempsey PC, Blankenship JM, Larsen RN (2017). Interrupting prolonged sitting in type 2 diabetes: nocturnal persistence of improved glycaemic control. Diabetologia.

[CR9] Dunstan DW, Kingwell BA, Larsen R (2012). Breaking up prolonged sitting reduces postprandial glucose and insulin responses. Diabetes Care.

[CR10] Duvivier B, Schaper NC, Koster A (2017). Benefits of substituting sitting with standing and walking in free-living conditions for cardiometabolic risk markers, cognition and mood in overweight adults. Front Physiol.

[CR11] Ekblom-Bak E, Bjorkman F, Hellenius ML, Ekblom B (2014). A new submaximal cycle ergometer test for prediction of VO_2max_. Scand J Med Sci Sports.

[CR12] Fratello F, Veniero D, Curcio G (2006). Modulation of corticospinal excitability by paired associative stimulation: reproducibility of effects and intraindividual reliability. Clin Neurophysiol.

[CR13] Gupta N, Hallman DM, Dumuid D, Vij A, Rasmussen CL, Jorgensen MB, Holtermann A (2020). Movement behavior profiles and obesity: a latent profile analysis of 24-h time-use composition among Danish workers. Int J Obes (Lond).

[CR14] Hothorn T, Bretz F, Westfall P (2008). Simultaneous inference in general parametric models. Biom J.

[CR15] Keel JC, Smith MJ, Wasserman EM (2000). A safety screening questionnaire for transcranial magnetic stimulation. Clin Neurophysiol.

[CR16] Kleim JA, Chan S, Pringle E, Schallert K, Procaccio V, Jimenez R, Cramer SC (2006). BDNF val66met polymorphism is associated with modified experience-dependent plasticity in human motor cortex. Nat Neurosci.

[CR17] Kujirai T, Caramia MD, Rothwell J (1993). Corticocortical inhibition in human motor cortex. J Physiol.

[CR18] Larsen R, Ali H, Dempsey PC (2019). Interrupting sitting time with simple resistance activities lowers postprandial insulinemia in adults with overweight or obesity. Obesity (Silver Spring).

[CR19] Lulic T, El-Sayes J, Fassett HJ, Nelson AJ (2017). Physical activity levels determine exercise-induced changes in brain excitability. PLoS ONE.

[CR20] Mang CS, Snow NJ, Campbell KL, Ross CJ, Boyd LA (2014). A single bout of high-intensity aerobic exercise facilitates response to paired associative stimulation and promotes sequence-specific implicit motor learning. J Appl Physiol (1985).

[CR21] McDonnell MN, Buckley JD, Opie GM, Ridding MC, Semmler JG (2013). A single bout of aerobic exercise promotes motor cortical neuroplasticity. J Appl Physiol (1985).

[CR22] Mellow ML, Goldsworthy MR, Coussens S, Smith AE (2019). Acute aerobic exercise and neuroplasticity of the motor cortex: a systematic review. J Sci Med Sport.

[CR23] Mooney RA, Coxon JP, Cirillo J, Glenny H, Gant N, Byblow WD (2016). Acute aerobic exercise modulates primary motor cortex inhibition. Exp Brain Res.

[CR24] Mullane SL, Buman MP, Zeigler ZS, Crespo NC, Gaesser GA (2016). Acute effects on cognitive performance following bouts of standing and light-intensity physical activity in a simulated workplace environment. J Sci Med Sport.

[CR25] Müller-Dahlhaus JF, Orekhov Y, Liu Y, Ziemann U (2008). Interindividual variability and age-dependency of motor cortical plasticity induced by paired associative stimulation. Exp Brain Res.

[CR26] Nooijen CFJ, Kallings LV, Blom V, Ekblom O, Forsell Y, Ekblom MM (2018). Common perceived barriers and facilitators for reducing sedentary behaviour among office workers. Int J Environ Res Public Health.

[CR27] Purves RD (1992). Optimum numerical integration methods for estimation of area-under-the-curve (AUC) and area-under-the-moment-curve (AUMC). J Pharmacokinet Biopharm.

[CR28] Ridding MC, Taylor JL (2001). Mechanisms of motor-evoked potential facilitation following prolonged dual peripheral and central stimulation in humans. J Physiol.

[CR29] Roig M, Skriver K, Lundbye-Jensen J, Kiens B, Nielsen JB (2012). A single bout of exercise improves motor memory. PLoS ONE.

[CR30] Sasaki JE, John D, Freedson PS (2011). Validation and comparison of ActiGraph activity monitors. J Sci Med Sport.

[CR31] Singh AM, Duncan RE, Neva JL, Staines WR (2014). Aerobic exercise modulates intracortical inhibition and facilitation in a nonexercised upper limb muscle. BMC Sports Sci Med Rehabil.

[CR32] Singh AM, Neva JL, Staines WR (2014). Acute exercise enhances the response to paired associative stimulation-induced plasticity in the primary motor cortex. Exp Brain Res.

[CR33] Smith AE, Goldsworthy MR, Garside T, Wood FM, Ridding MC (2014). The influence of a single bout of aerobic exercise on short-interval intracortical excitability. Exp Brain Res.

[CR34] Stefan K, Kunesch E, Cohen LG, Benecke R, Classen J (2000). Induction of plasticity in the human motor cortex by paired associative stimulation. Brain.

[CR35] Stefan K, Kunesch E, Benecke R, Cohen LG, Classen J (2002). Mechanisms of enhancement of human motor cortex excitability induced by interventional paired associative stimulation. J Physiol.

[CR36] Stefan K, Wycislo M, Classen J (2004). Modulation of associative human motor cortical plasticity by attention. J Neurophysiol.

[CR37] van Praag H, Christie BR, Sejnowski TJ, Gage FH (1999). Running enhances neurogenesis, learning, and long-term potentiation in mice. Proc Natl Acad Sci USA.

[CR38] Witte AV, Kurten J, Jansen S, Schirmacher A, Brand E, Sommer J, Floel A (2012). Interaction of BDNF and COMT polymorphisms on paired-associative stimulation-induced cortical plasticity. J Neurosci.

