# Supplementary material for: The effect of breaking up prolonged sitting on paired associative stimulation-induced plasticity
Source: Exp Brain Res. 2020 Aug 28;238(11):2497–506. doi: 10.1007/s00221-020-05866-z (PMC7541377; doi:10.1007/s00221-020-05866-z)
Supplement: Supplementary file 1 — Supplementary file1 (DOCX 13 kb) [file 221_2020_5866_MOESM1_ESM.docx]

# Supplemental material

| **Table 4**  Pre-planned pairwise comparisons for time | | | |
| --- | --- | --- | --- |
|  | β | Std.Error | *p-*value |
| Baseline – Post.int | -0.045 | 0.029 | 0.405 |
| Baseline – Post.PAS.5 | -0.077 | 0.030 | 0.053 |
| Baseline – Post.PAS.30 | -0.113 | 0.030 | 0.001* |
| Post.int – Post.PAS.5 | -0.032 | 0.030 | 0.466 |
| Post.int – Post.PAS.30 | -0.068 | 0.030 | 0.093 |
| Post.PAS.5 – Post.PAS.30 | -0.036 | 0.030 | 0.466 |

| **Table 5**  Pre-planned pairwise comparisons for each experimental condition | | | |
| --- | --- | --- | --- |
| SIT | β | Std.Error | *p-*value |
| Baseline – Post.int | -0.019 | 0.052 | 1.000 |
| Baseline – Post.PAS.5 | -0.036 | 0.052 | 1.000 |
| Baseline – Post.PAS.30 | -0.081 | 0.052 | 1.000 |
| Post.int – Post.PAS.5 | -0.017 | 0.052 | 1.000 |
| Post.int – Post.PAS.30 | -0.063 | 0.052 | 1.000 |
| Post.PAS.5 – Post.PAS.30 | -0.045 | 0.052 | 1.000 |
| FPA |  |  |  |
| Baseline – Post.int | -0.123 | 0.050 | 0.233 |
| Baseline – Post.PAS.5 | -0.158 | 0.051 | 0.037* |
| Baseline – Post.PAS.30 | -0.184 | 0.051 | 0.006* |
| Post.int – Post.PAS.5 | -0.034 | 0.051 | 1.000 |
| Post.int – Post.PAS.30 | -0.061 | 0.051 | 1.000 |
| Post.PAS.5 – Post.PAS.30 | -0.026 | 0.052 | 1.000 |
| EXE |  |  |  |
| Baseline – Post.int | 0.009 | 0.050 | 1.000 |
| Baseline – Post.PAS.5 | -0.036 | 0.050 | 1.000 |
| Baseline – Post.PAS.30 | -0.073 | 0.050 | 1.000 |
| Post.int – Post.PAS.5 | -0.046 | 0.050 | 1.000 |
| Post.int – Post.PAS.30 | -0.082 | 0.050 | 1.000 |
| Post.PAS.5 – Post.PAS.30 | -0.036 | 0.050 | 1.000 |

Pre-planned pairwise comparisons for each experimental condition. SIT = prolonged sitting, FPA = breaking up prolonged sitting by frequent short bouts of physical activities. EXE = prolonged sitting followed by an exercise bout
